# Setting the stage for cardiomyopathy gene editing trials: a systematic review of isogenic pair use in human induced pluripotent stem cell-derived cardiomyocyte research

**DOI:** 10.1093/ehjopen/oeaf161

**Published:** 2025-12-03

**Authors:** C Nina van der Wilt, Rogier J A Veltrop, Maaike H Janssens, Iara Bakker, Francesca Stillitano, Joost P G Sluijter, Linda W van Laake, Jolanda van der Velden, Eric Villard, Judith Montag, Chris Denning, J Peter van Tintelen, Anneline S J M te Riele, Pim van der Harst, Leon J Schurgers, Frank G van Steenbeek, Magdalena Harakalova

**Affiliations:** Department of Cardiology, Division of Heart and Lungs, University Medical Center Utrecht, Utrecht University, Heidelberglaan 100, 3584 CX Utrecht, The Netherlands; Regenerative Medicine Center Utrecht and Circulatory Health Research Center, University Medical Center Utrecht, Utrecht University, Heidelberglaan 8, 3584 CS Utrecht, The Netherlands; Department of Cardiology, Division of Heart and Lungs, University Medical Center Utrecht, Utrecht University, Heidelberglaan 100, 3584 CX Utrecht, The Netherlands; Regenerative Medicine Center Utrecht and Circulatory Health Research Center, University Medical Center Utrecht, Utrecht University, Heidelberglaan 8, 3584 CS Utrecht, The Netherlands; Department of Biochemistry, CARIM, Maastricht University, Universiteitssingel 50, 6229 ER Maastricht, The Netherlands; LMNAcardiac.org, LMNA Patient Organization, Vredehofstraat 13761, HA Soest, The Netherlands; Netherlands Heart Institute, Holland Heart House, Moreelsepark 1, 3511 EP Utrecht, The Netherlands; Department of Cardiology, Division of Heart and Lungs, University Medical Center Utrecht, Utrecht University, Heidelberglaan 100, 3584 CX Utrecht, The Netherlands; Regenerative Medicine Center Utrecht and Circulatory Health Research Center, University Medical Center Utrecht, Utrecht University, Heidelberglaan 8, 3584 CS Utrecht, The Netherlands; Department of Cardiology, Division of Heart and Lungs, University Medical Center Utrecht, Utrecht University, Heidelberglaan 100, 3584 CX Utrecht, The Netherlands; Regenerative Medicine Center Utrecht and Circulatory Health Research Center, University Medical Center Utrecht, Utrecht University, Heidelberglaan 8, 3584 CS Utrecht, The Netherlands; Department of Cardiology, Division of Heart and Lungs, University Medical Center Utrecht, Utrecht University, Heidelberglaan 100, 3584 CX Utrecht, The Netherlands; Regenerative Medicine Center Utrecht and Circulatory Health Research Center, University Medical Center Utrecht, Utrecht University, Heidelberglaan 8, 3584 CS Utrecht, The Netherlands; Netherlands Heart Institute, Holland Heart House, Moreelsepark 1, 3511 EP Utrecht, The Netherlands; Department of Cardiology, Division of Heart and Lungs, University Medical Center Utrecht, Utrecht University, Heidelberglaan 100, 3584 CX Utrecht, The Netherlands; Regenerative Medicine Center Utrecht and Circulatory Health Research Center, University Medical Center Utrecht, Utrecht University, Heidelberglaan 8, 3584 CS Utrecht, The Netherlands; Department of Cardiology, Division of Heart and Lungs, University Medical Center Utrecht, Utrecht University, Heidelberglaan 100, 3584 CX Utrecht, The Netherlands; Regenerative Medicine Center Utrecht and Circulatory Health Research Center, University Medical Center Utrecht, Utrecht University, Heidelberglaan 8, 3584 CS Utrecht, The Netherlands; Department of Physiology, Amsterdam Cardiovascular Sciences, Amsterdam University Medical Center, De Boelelaan 1108, 1081 HZ Amsterdam, The Netherlands; Sorbonne Université, INSERM, UMRS 1166, 47-83, Boulevard de l'Hôpital, 75013 Paris, France; APHP, Pitié-Salpêtrière University Hospital, 47-83 Boulevard de l'Hôpital, 75013 Paris, France; ICAN Biocell iPS Core – Institute for Cardiometabolism and Nutrition, 47-83 Boulevard de l'Hôpital, 75013 Paris, France; Department of Human Medicine, Medical School Berlin, Rüdesheimer Str. 50, 14197 Berlin, Germany; Biodiscovery Institute, University of Nottingham, Life Sciences Building University Park, NG7 2RD Nottingham, UK; Department of Genetics, University Medical Center Utrecht, Utrecht University, Heidelberglaan 100, 3584 CX Utrecht, The Netherlands; Department of Cardiology, Division of Heart and Lungs, University Medical Center Utrecht, Utrecht University, Heidelberglaan 100, 3584 CX Utrecht, The Netherlands; Department of Cardiology, Division of Heart and Lungs, University Medical Center Utrecht, Utrecht University, Heidelberglaan 100, 3584 CX Utrecht, The Netherlands; Department of Biochemistry, CARIM, Maastricht University, Universiteitssingel 50, 6229 ER Maastricht, The Netherlands; Department of Cardiology, Division of Heart and Lungs, University Medical Center Utrecht, Utrecht University, Heidelberglaan 100, 3584 CX Utrecht, The Netherlands; Regenerative Medicine Center Utrecht and Circulatory Health Research Center, University Medical Center Utrecht, Utrecht University, Heidelberglaan 8, 3584 CS Utrecht, The Netherlands; Department of Clinical Sciences, Faculty of Veterinary Medicine, Utrecht University, Yalelaan 104-106, 3584 CM Utrecht, The Netherlands; Department of Cardiology, Division of Heart and Lungs, University Medical Center Utrecht, Utrecht University, Heidelberglaan 100, 3584 CX Utrecht, The Netherlands; Regenerative Medicine Center Utrecht and Circulatory Health Research Center, University Medical Center Utrecht, Utrecht University, Heidelberglaan 8, 3584 CS Utrecht, The Netherlands; Department of Biochemistry, CARIM, Maastricht University, Universiteitssingel 50, 6229 ER Maastricht, The Netherlands; Netherlands Heart Institute, Holland Heart House, Moreelsepark 1, 3511 EP Utrecht, The Netherlands

**Keywords:** CRISPR-Cas, Genome editing, Variant pathogenicity, Standardized clinical reporting, Clinical trials

## Abstract

*In vitro* gene editing using isogenic pairs of human induced pluripotent stem cell-derived cardiomyocytes (hiPSC-CMs) has demonstrated the feasibility of introducing or correcting specific pathogenic variants. These successes represent a key first step towards therapeutic genome editing for cardiomyopathies, showing that precise, variant-specific interventions are achievable. To translate *in vitro* findings to the clinic, it is essential to develop robust disease models that yield meaningful, translatable data. The next challenge is systematically identifying disease-causing variants amenable to gene editing with strong pre-clinical support. Therefore, we conducted a systematic search of published studies on isogenic hiPSC-CM pairs in cardiomyopathy research with specific criteria, including (likely) pathogenic variants causing cardiomyopathy, correction and/or introduction of variants, differentiation into CMs, and functional follow-up. We systematically assessed 785 papers and highlighted 101 studies meeting our inclusion criteria reporting 69 patients carrying 56 unique variants across 31 genes, most commonly *MYH7*, *MYBPC3*, and *DMD*. This expanded to 91 variants across 38 genes upon inclusion of the introduced variants in a donor line. However, reported clinical data were often incomplete, underscoring the need for standardized phenotypic documentation. We reveal a lack of patient details, which creates an incomplete picture of underlying disease variables that hinder the design of targeted personalized treatments. Omitted key clinical data can lead to misinterpretations or overlooked variables that impact treatment outcomes. This systematic review integrates current evidence from successful *in vitro* studies using isogenic hiPSC-CM models and proposes a reporting framework for variant prioritization and the rigorous application of isogenic controls in cardiomyopathy research.

## Graphical Abstract

**Graphical Abstract:**
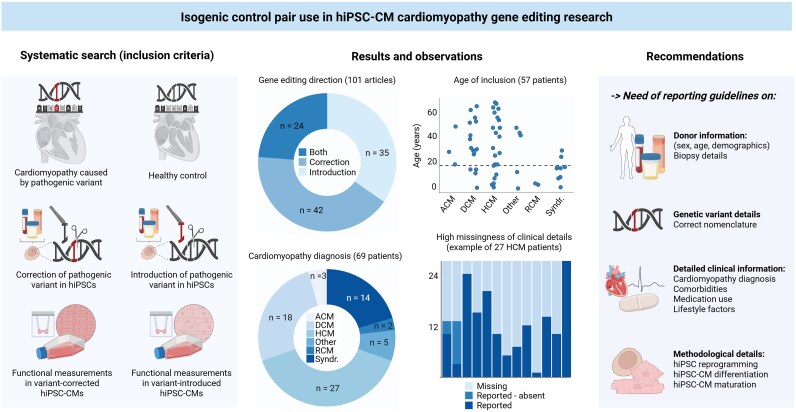

## Basic principles of human induced pluripotent stem cell-based models used in cardiomyopathy research

Cardiomyopathies are characterized as myocardial disorders in which the heart muscle is structurally and functionally abnormal, in the absence of coronary artery disease (CAD), hypertension, valvular disease, or congenital heart disease (CHD). These abnormalities form a diverse group that can have isolated and syndromic forms. Isolated cardiomyopathies, characterized as hypertrophic cardiomyopathy (HCM), dilated cardiomyopathy (DCM), arrhythmogenic (right ventricular) cardiomyopathy (ACM/ARVC), restrictive cardiomyopathy (RCM), and non-dilated left ventricular (LV) cardiomyopathy (NDLVC), are far more common than syndromic cardiomyopathies (e.g. associated with Noonan syndrome, Barth syndrome, or Duchenne muscular dystrophy).^1^ Cardiomyopathies account for ∼50% of all heart transplants (HTx), 40% of long-term mechanical circulatory supports, and 30% of defibrillator implantations.^2^ Current treatments, besides HTx, are supportive and may alleviate symptoms. There are even disease-modifying therapies for cardiac amyloidosis, Fabry disease, and sarcomeric HCM (myosin inhibitors).^3–5^ Yet, to date, fully curative treatments and personalized treatments are lacking for cardiovascular diseases. To alleviate the disease burden, a further understanding of the pathophysiological mechanisms underlying these diseases is needed to identify possible therapeutic targets and specific biomarkers at an individual level.^6,7^ Pathogenic and likely pathogenic variants in over a hundred genes have been proposed to be associated with genetic cardiomyopathies.^8,9^ However, many of these genes lack strong evidence for a causal relationship with disease. As a result, only a curated subset of genes with well-established gene–disease associations is currently included in standard diagnostic testing panels.^10,11^ These genes encode proteins essential for the structural integrity and functionality of cardiomyocytes (CMs). Consequently, but not exclusively, research should focus on CMs, the primary cardiac cell type impacted in cardiomyopathies.

There are several options when it comes to studying CMs using the patient’s cardiac tissue. A myocardial biopsy may, in distinct cases such as fulminant or unexplained heart failure (HF) and suspected giant cell or eosinophilic myocarditis, provide critical insights into the disease characteristics and may aid in rapid diagnosis when immediate treatment is required. However, it is an invasive procedure that carries potential risks, including sampling bias, perforation, and damage to the heart valves.^12^ Additionally, cardiac biopsy allows for a minimal amount of the myocardium to be studied, which is often taken from the myocardial septum and might therefore not represent the ventricular phenotype in several cardiomyopathies.^13^ Cardiac tissue obtained during procedures for ventricular assist device (VAD) implantation or after HTx can be used to obtain CMs. However, culturing primary CMs *in vitro* presents a challenge since they are non-proliferative and can only be cultured for a few days.^14^ Recent advances in fresh heart slice culturing allow for experiments over a few weeks, enabling functional testing. However, obtaining this type of cardiac tissue is mostly possible at progressive cardiomyopathy stages, in cases of HTx or VAD implantations.^15^ Even with careful progress in the field, the heart still lacks a readily available source of functional somatic adult stem cells that can be isolated and reliably used to produce cardiac organoids, unlike most other organs.^16^ Hence, the development of protocols to culture human induced pluripotent stem cells (hiPSCs)^17^ and subsequent differentiation into CMs provides a platform for high-throughput cellular experiments on cells partially resembling the adult CM, while retaining a foetal stage of maturation.^18^ hiPSC reprogramming can be performed using different methods, including an episomal vector or a Sendai virus expressing reprogramming factors like POU5F1 (OCT4), SOX2, KLF4, and MYC.^17^ hiPSCs can be generated from non-cardiac patient sources, such as blood, skin, or urine, and differentiated into various specialized cell types, including CMs.^19^ This pipeline offers a versatile and renewable source for creating functional cardiac models, amendable to genetic modification.

The initial step of a hiPSC experiment, the collection of source cells used for reprogramming, should be tightly coordinated with clinics for several reasons. The first hiPSC experiments were performed with dermal fibroblasts (DFs) obtained by skin biopsy, typically under local anaesthesia.^20^ Despite being relatively easy to culture *in vitro*, the fibroblast post-cryopreservation viability and proliferation rate depend on the biopsy site.^21^ A few years later, peripheral blood mononuclear cells (PBMCs) became more commonly used because they produce hiPSC-derive CMs (hiPSC-CMs) with features similar to those derived from DFs while being more practical and less invasive to obtain.^22^ Performing PBMC isolation shortly after the blood draw is crucial, as PBMC quality decreases the longer the time between the blood draw and isolation.^23^ The use of anaesthetics has a major impact on PBMC quality as well, as it causes a certain degree of hypoxia and PBMC damage.^23,24^ Notably, the reprogramming is not adversely affected when performed on PBMCs extracted from elderly patients,^25^ in contrast with DFs.^26^ A more recent novel reprogramming method of generating hiPSCs from urine opened new avenues through its non-invasive nature in the collection process.^27^ Therefore, careful coordination with clinics is essential to ensure the viability and functionality of the collected cells, considering patient-specific factors that may affect cell quality. The process must be fully consented to and integrated with a functional biobanking workflow to ensure ethical handling and long-term preservation of samples.^28^

## Isogenic human induced pluripotent stem cell-derived cardiomyocyte control pair use as a strategy to test genome editing consequences

*In vitro* gene editing using isogenic pairs of hiPSCs has demonstrated the feasibility of introducing or correcting specific pathogenic variants. These successful applications represent a critical first step towards therapeutic genome editing for cardiomyopathies, showing that precise, variant-specific interventions can be achieved at the genomic level. To move from bench to bedside, however, we must not only prove that editing is technically possible but also establish robust disease models that generate meaningful, translatable data. In this context, isogenic hiPSC pairs consisting of mutant and wild-type lines play an important role in dissecting the molecular pathobiology of cardiomyopathies and assessing functional consequences of individual variants. These controlled *in vitro* systems enable direct attribution of phenotypic differences to specific genetic mutations, thereby increasing confidence in variant pathogenicity and therapeutic relevance.

Isogenic controls of a hiPSC line are defined as lines with the same genetic background, where the studied variant is corrected or introduced. In case of an introduction of a variant on a healthy donor background, any differences between the control and variant-induced hiPSC lines are expected to occur exclusively due to the studied DNA variant and not to genetic background effects, penetrance, or expressivity.^29^ On the other hand, correcting a pathogenic variant in a patient-derived hiPSC line offers a uniquely valuable approach, as it demonstrates that the phenotype can be rescued despite the individual’s complex genetic background, making it especially relevant for personalized medicine and studying variant expressivity in diverse genetic contexts.^30^ Furthermore, a homozygous mutation or knock-out (KO) can be introduced to validate if the disease phenotype worsens compared to the mostly occurring heterozygous disease phenotype.^31,32^ Gene (genome) editing can be applied not only in the hiPSC stage prior to differentiation into the desired cell type but also in the differentiated desired cell type. Over the past decades, numerous gene editing tools have been developed. These techniques are based on genetic principles using zinc finger nuclease (ZFN), transcription activator-like effector nuclease (TALEN), homologous recombination (HR), clustered regularly interspaced short palindromic repeats–CRISPR-associated protein (CRISPR-Cas), adenine base editing (ABE), and prime editing (PE).^29,33–38^ Currently, the most commonly used techniques for hiPSC gene editing are CRISPR-Cas-based approaches.

hiPSCs of an isogenic pair can subsequently be differentiated into cardiac cells. The differentiation from hiPSC to hiPSC-CMs is based on known developmental stimuli such as endoderm co-culture, growth factor signalling, and biological manipulation using small molecules. Frequently used additives include Activin/Nodal, TGFB, GSK3, Wnt, and BMP.^39–41^ hiPSC-CMs have a relatively immature phenotype that resembles foetal or neonatal CMs in terms of cell size and morphology, gene expression, myofibril contractility, and metabolic activity.^42–45^ To overcome this limitation, various methods have been developed to mature hiPSC-CMs, including prolonged culture, the use of a fatty acid-rich medium,^46^ culturing on nanopatterned surfaces,^47^ engineered heart tissue (EHT),^48^ electromechanical EHT stimulation,^47^ and the creation of three-dimensional (3D) microtissues composed of multiple cell types.^42,49^ Such setups can be used to study cellular characteristics such as electrical activity and action potentials, calcium handling, and contractility. These assays are suitable for *in vitro* disease modelling and drug discovery and for quantifying and characterizing the effects of gene editing at a cellular level.

While hiPSC-CM technology has advanced significantly, it remains a highly complex process that requires specialized expertise and skilled personnel to carry out successfully. The advantages of hiPSCs include eliminating the need for myocardial biopsies, genetic capacity to differentiate into all three germ layers,^32^ compatibility with high-throughput settings, their ability to recapitulate phenotypic characteristics caused by genetic variations, and the possibility for 3D tissue modelling.^50^ Another advantage is that they are of human origin, offering a key benefit over small animals, which are most often used to model cardiovascular disease but carry important differences compared to their human counterparts. These differences include heart rate, cell size, multinucleation frequency, and myosin heavy chain expression.^43^ It has to be noted that while the hiPSC-CM models are being continuously improved, the use of animal models remains important for evaluating translational aspects related to immunogenicity, biodistribution, and long-term effects of therapies. On the other hand, limitations of hiPSC-CM technology include their relatively immature phenotype, heterogeneity between batches of hiPSC-CM differentiation, variable presence of non-myocytes in culture, residual somatic epigenetic signature, and the fact that, in the case of an isogenic control pair setup, they cannot be used for disease modelling of conditions with an unknown genetic aetiology.^14^ It is important to note that comparable protein expression and thus function in diseased and control hiPSC-CMs rely on standardized culture protocols to decrease heterogeneity. The profiles of mRNA and protein expression and functional readouts change back to a more immature stage upon detachment and replating, requiring ∼1 week to recover to a more mature stage.^51^

## From dish to bedside

hiPSC-CMs are typically used for *in vitro* disease modelling, such as electrophysiological and toxicological experiments for either basic research or personalized translational research.^52–54^ These experiments also show promise for regenerative therapeutic options, including gene therapy and gene (genome) editing.^53,55^ So far, *in vivo* gene editing has only been successfully performed in animal cardiomyopathy models^56–58^ and in humans by addressing organs that are easily accessible, such as blood,^59^ skin,^60^ and the eye.^61^ Phase 1 study involving patients with transthyretin amyloid cardiomyopathy treated with intravenous infusion of nexiguran ziclumeran, gene editing therapy based on CRISPR-Cas9 targeting the transthyretin gene (*TTR*), was associated with reductions in serum TTR levels.^62^ A single *in vivo* administration of lipid nanoparticle-delivered CRISPR base editors durably and precisely edited the PCSK9 gene in cynomolgus monkeys as well, leading to sustained large reductions in PCSK9 and LDL cholesterol, providing a proof of concept for a promising gene editing approach to treat atherosclerotic cardiovascular disease.^63^ While gene editing is typically applied in the hiPSC stage before differentiation, novel PE protocols enable the correction of variants directly in hiPSC-CMs.^64^ Despite progress in the field, an overarching effective technology for delivering genome therapy is still under development for the heart.^65^ Nonetheless, as the field transitions from comparing readouts between isogenic control lines towards clinical trials and implementation of gene editing in patient care,^66^ it is essential to have a clear overview of which genes and variants have been targeted. In addition, experimental information needs to be coupled with details of the sampling source of cells for hiPSC reprogramming, as well as the demographic and clinical information of the patients involved in gene editing experiments.

Inclusion of patient-specific factors is particularly essential for several reasons. Patient age, disease stage, and genetic background^67–69^ may significantly influence the differentiation potential of hiPSC-CMs or their response to gene editing, making it difficult to predict therapeutic efficacy in different patient populations. Without this context, it is challenging to know whether findings from one patient cohort will apply to others, potentially limiting the broader applicability of gene therapy approaches. Furthermore, given the heterogeneity of cardiomyopathy progression,^70^ a lack of patient details in current studies may create an incomplete picture of the disease mechanisms and hinder the design of targeted personalized treatments. Omitting key clinical data can lead to misinterpretations or overlooked variables that impact treatment outcomes.^71^

Previous reports have typically focused on the technology itself, the successful correction of the variant, or the analysis of readouts^14,72,73^ rather than incorporating the clinical information about the patients who donated their cells for hiPSC reprogramming. Therefore, we systematically reviewed the current literature on isogenic control pairs in cardiomyopathy research to identify genetic variants for which gene editing has been performed *in vitro* and evaluate the reporting of the details of practical demographic and clinical parameters important to enhance clinical translation.

## Systematic overview

**Figure 1:**
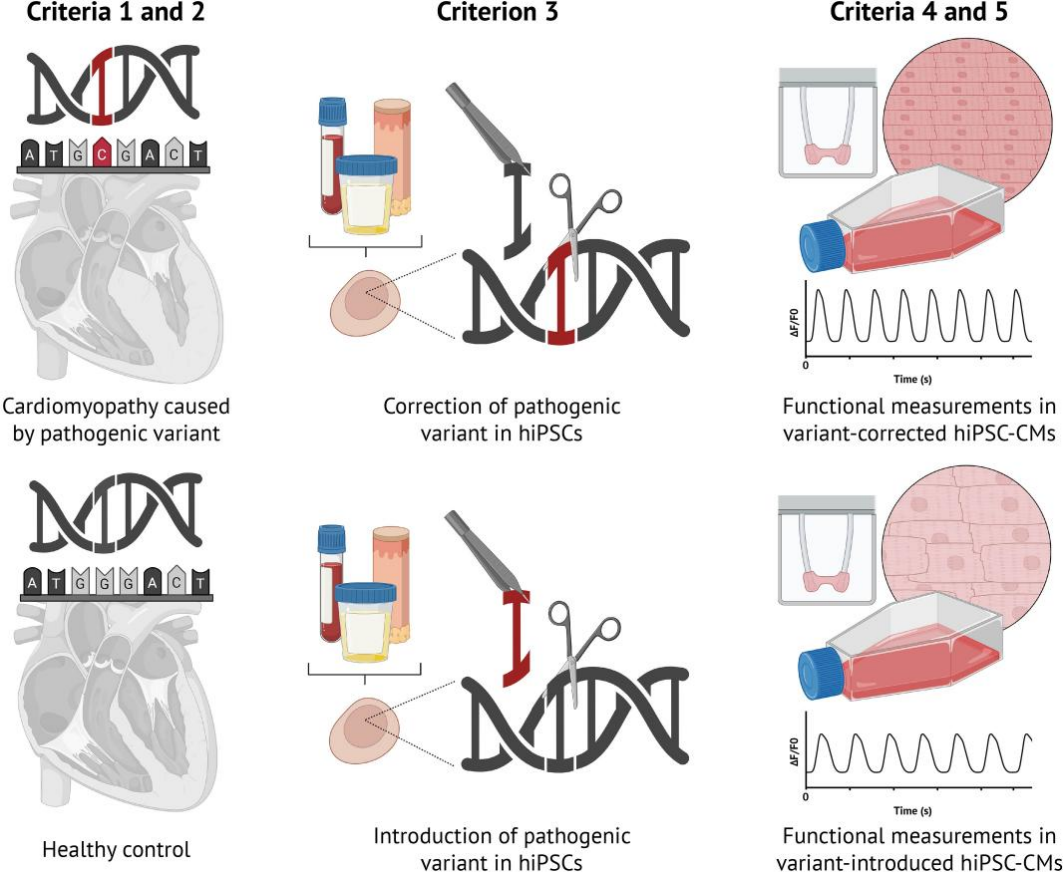
Schematic overview of the systematic search on PubMed. A systematic search was performed on PubMed Advanced Search Builder. All resulting articles were checked and scored according to five selection criteria: (i) reported human (pathogenic) variants associated with a patient cardiomyopathy phenotype either in the cell-donating patient (variant correction) or in previously reported patients (variant introduction); (ii) cardiomyopathy diagnosed clinically and caused by a pathogenic variant either in the cell-donating patient (variant correction) or in previously reported patients (variant introduction); (iii) correction and/or introduction of the pathogenic variant in a human induced pluripotent stem cell line using gene editing; (iv) differentiation into cardiomyocytes (human induced pluripotent stem cell-derived cardiomyocytes); and (v) functional measurements in the differentiated human induced pluripotent stem cell-derived cardiomyocyte isogenic pair, including live cell measurements, such as electrophysiology, contractility, or calcium handling. Figure created using BioRender (https://biorender.com/).

**Figure 2:**
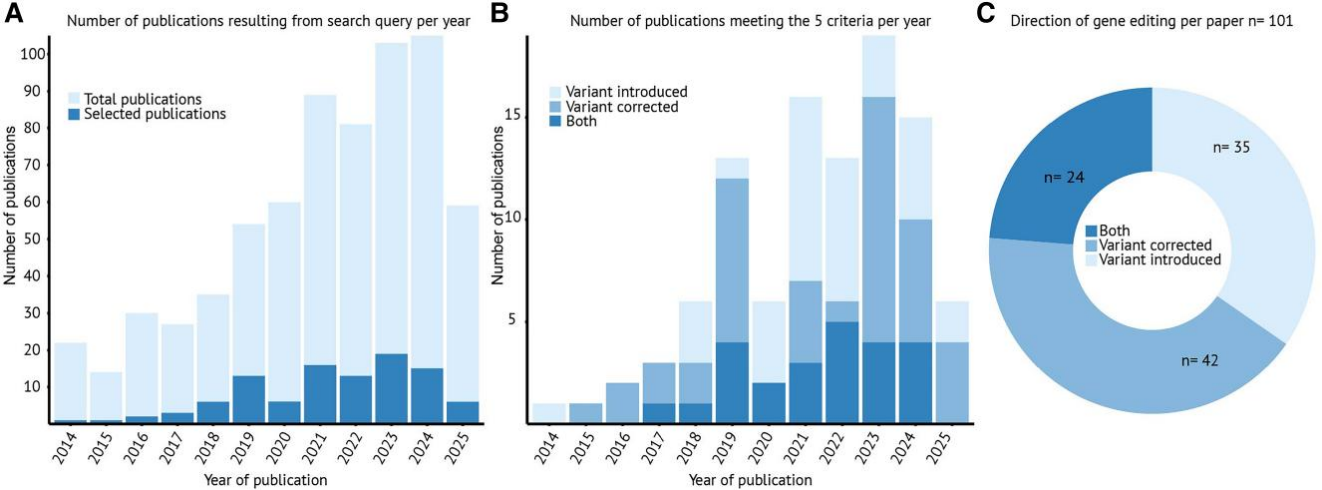
Characteristics of the investigated articles dealing with the use of isogenic controls in cardiomyopathy research. PubMed database was searched for publications on human induced pluripotent stem cell-derived cardiomyocytes used for research on cardiomyopathies, which provide isogenic controls. (*A*) Total count of publications and count of publications that match the five criteria of this review. The search was conducted on 25 August 2025. (*B*) Subdivision of publications matching the inclusion criteria into publications using only introduced disease-causing variants and donor isogenic controls, using only patient-derived human induced pluripotent stem cells and isogenic controls generated by correction of the mutation, and using both patient-derived and introduced disease-causing human induced pluripotent stem cells and the respective isogenic controls. (*C*) Total of included papers subdivided by three gene editing strategies: introducing the pathogenic variant into healthy human induced pluripotent stem cells, correcting the pathogenic variant in patient-derived human induced pluripotent stem cells, or both.

We performed a systematic PubMed search to identify available studies on the use of isogenic control pairs through *in vitro* gene editing for cardiomyopathy research. See Supplementary Methods for more details. Five inclusion criteria were used: (i) reported human (pathogenic) variants associated with a patient cardiomyopathy phenotype either in the cell-donating patient (variant correction) or in previously reported patients (variant introduction); (ii) cardiomyopathy diagnosed clinically and caused by a pathogenic variant either in the cell-donating patient (variant correction) or in previously reported patients (variant introduction); (iii) correction and/or introduction of the pathogenic variant in a hiPSC line using gene editing; (iv) differentiation into CMs (hiPSC-CMs); and (v) functional measurements in the differentiated hiPSC-CM isogenic pair, including live cell measurements, such as electrophysiology, contractility, or calcium handling (*Figure 1*). The results were processed following the PRISMA protocol^74^ (see Supplementary material online, *Figure S1*). The search yielded 785 papers, of which 101 (12.8%) met the five inclusion criteria (see Supplementary material online, *Table S1*). The number of publications per year shows an increasing trend, with the first publication reported in 2014. There is an evident dip in publication records during 2020 and 2022, likely due to the consequences of the COVID-19 pandemic (*Figure 2A*). In 35 of the included papers, only healthy (donor) hiPSCs were used to introduce a pathogenic DNA variant (mutation). The remaining 66 papers involved patients with corrected hiPSCs or healthy cells that had a variant introduced and were then corrected; 42 papers focused solely on correction and not introduction, while 24 included both correction and introduction of disease-causing variants (*Figure 2B* and *C*). Notably, three studies introduced cardiomyopathy variants in a donor line and subsequently corrected them.^64,75,76^ These findings point to the increasing research efforts and the use of various approaches in creating isogenic control hiPSC-CM pairs for genetic editing in cardiomyopathy studies.

**Figure 3:**
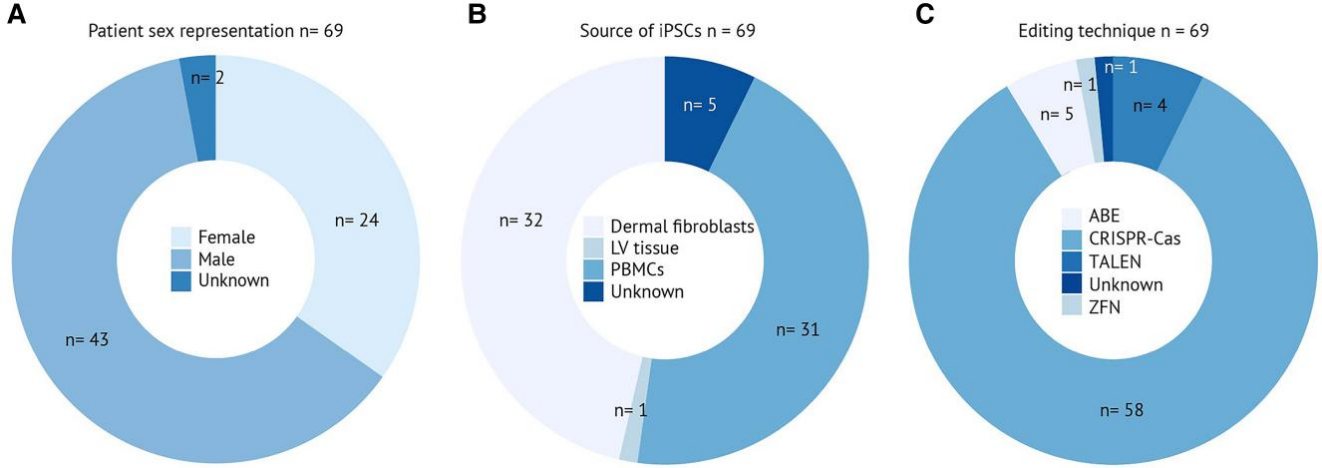
Characteristics of identified cardiomyopathy patients whose cells were subjected to human induced pluripotent stem cell reprogramming and human induced pluripotent stem cell-derived cardiomyocyte differentiation, including the creation of an isogenic control (correction of the variant). (*A*) Sex distribution of the donating patient cohort. (*B*) Source of cells for human induced pluripotent stem cell generation, including dermal fibroblasts, left ventricular tissue, peripheral blood mononuclear cells, and cases with unknown origins. (*C*) Gene editing techniques used for the generation of isogenic controls, including adenine base editing, clustered regularly interspaced short palindromic repeats–CRISPR-associated protein, transcription activator-like effector nuclease, and zinc finger nuclease technologies.

There were 69 cardiomyopathy patients included in the 64 studies focusing on correcting the original pathogenic variant in patient hiPSCs. The male-to-female ratio was 64.2%:35.8% (*Figure 3A*), consistent with the higher prevalence of cardiomyopathy observed in males in clinical populations,^1^ reflecting the importance of including both sexes to capture the full spectrum of disease phenotypes. Information on sex of the included patients was unclear in two cases. The hiPSCs were derived from different sources. The most common sources were PBMCs (44.9%) and DFs (46.4%), and from one patient, cells were retrieved from the left ventricle (1.4%). Hence, the source was not clearly specified for five patients in two studies (9.2%) (*Figure 3B*). Gene editing strategies included CRISPR-Cas (85.5%), ABE (7.2%), TALEN (5.8%), and ZFN (1.4%) (*Figure 3C*), emphasizing the reliance on CRISPR-Cas as the predominant gene editing approach. The variability in cell sources and editing techniques highlights the need for standardized protocols for better reproducibility and comparability of results in future cardiomyopathy research.

**Figure 4:**
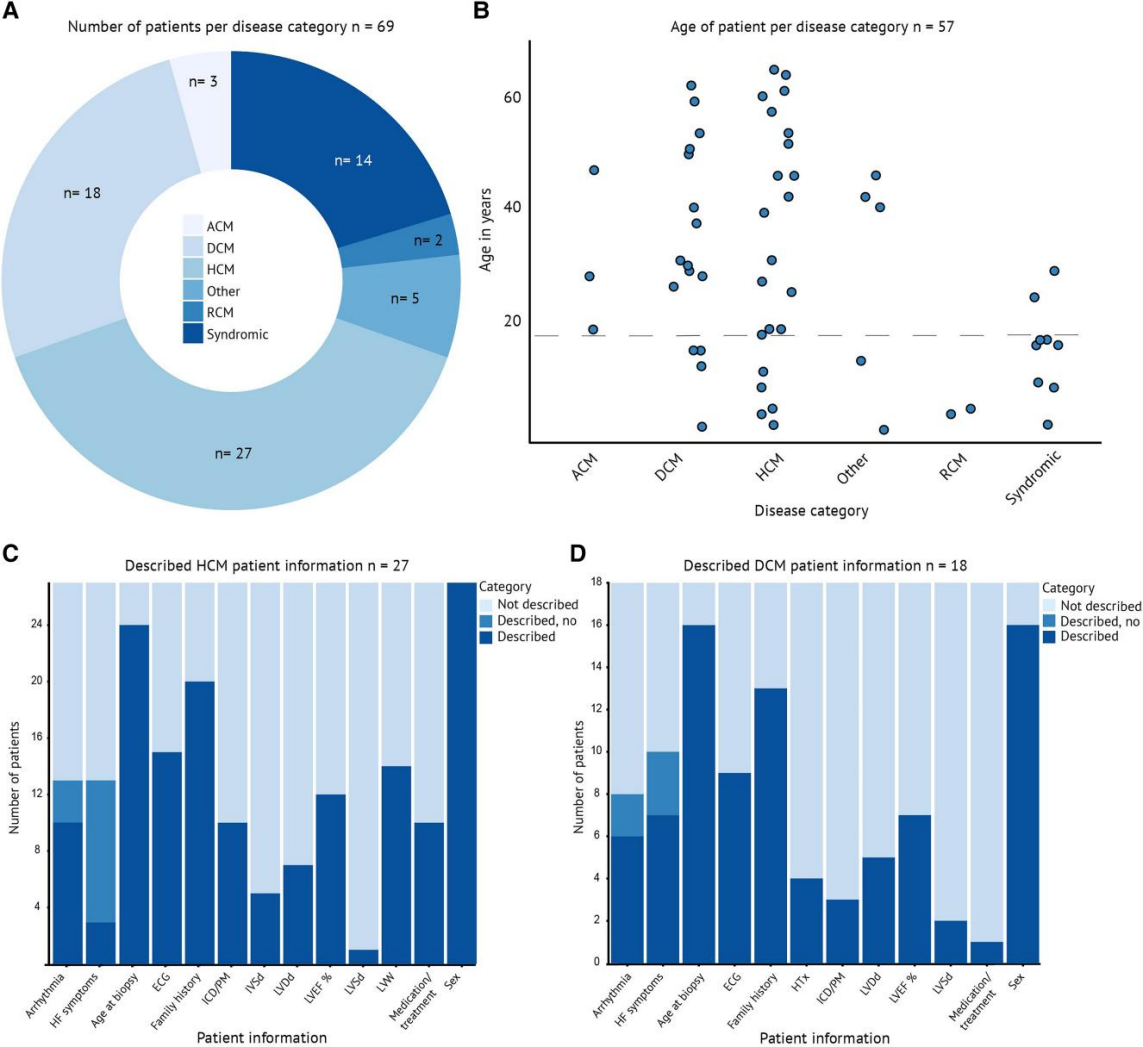
Information on disease and patients given in the publications. (*A*) Number of patients per disease category. (*B*) Age of patients at sampling per disease category. For two patients where only an age range was provided, these were omitted from the plot. (*C*) Number of hypertrophic cardiomyopathy patients for whom specific clinical information was described (dark blue) or not described (light blue). (*D*) Number of dilated cardiomyopathy patients for whom specific clinical information was described (dark blue) or not described (light blue). ECG, electrocardiogram; HF, heart failure; HTx, heart transplant; ICD, implantable cardioverter defibrillator; PM, pacemaker; IVSd, interventricular septum diameter; LVDd, left ventricular diastolic diameter; LVEF, left ventricular ejection fraction; LVSd, left ventricular systolic diameter; LVW, left ventricular wall.

Cardiomyopathy is an umbrella term for diseases with various aetiologies. To identify the cardiomyopathy subtypes for which isogenic control pairs for *in vitro* gene editing have already been established, we grouped disease types reported in the 64 studies reporting a correction of a variant associated with cardiomyopathy. Where applicable, patients were assigned to cardiomyopathy subgroups based on the European Society of Cardiology (ESC) Clinical Practice Guidelines.^1^ For isolated forms of cardiomyopathies, the results show that 39.1% of the total patients had HCM, followed by 26.1% with DCM, 4.3% with ACM, and 2.9% with RCM. Notably, no patient met the criteria for the recently described NDLVC subtype of cardiomyopathy. NDLVC is a novel cardiomyopathy diagnosis that is characterized by non-ischaemic LV myocardial fibrosis or fibrofatty remodelling without LV dilatation or systolic dysfunction.^1^ Cardiomyopathies due to syndromic disease were present in 20.3% of identified patients. This group included Danon disease, Noonan syndrome, Friedreich’s ataxia, Fabry disease, and Duchenne muscular dystrophy. This last group is noteworthy as gene editing strategies for patients’ cells focused on correcting deletions of whole exons instead of a single nucleotide variant (5.8% of total patients). Finally, four patients with a LV non-compaction (LVNC) phenotype and another with myocarditis made up a collective 7.2% of patients and were classified as ‘Other’ (*Figure 4A*). This analysis offers an overview of the cardiomyopathy subtypes studied and underscores gaps in current hiPSC-CM isogenic control pair research, such as for NDLVC, ACM, LVNC, or rare forms of syndromic cardiomyopathies.

The age at sampling, which was included for 85.5% of the total identified patients, was plotted per diagnosis in *Figure 4B*. The data of all patients whose hiPSCs were corrected show that successful hiPSC generation and correction of a (supposed) pathogenic variant are possible in hiPSCs generated from patients of all ages, starting from cells collected from patients younger than 1 year to a patient at the age of 68 years. Although symptoms of most cardiomyopathies only present later in life and despite ethical constraints regarding age in participation in clinical studies, 35.6% of the patient cohort was <18 years at the age of sampling or diagnosis. Notably, the onset of disease in HCM patients typically shows a mean of around 40 years but peaks at the first year of life, around the teenage years, and around 50 years of age.^77^ Five of the reported HCM patients whose ages were disclosed were <18 years old (17.9%), with the youngest identified HCM patient not yet 1 year old, while the oldest HCM patient had reached the age of 68 at the time of sampling. ACM typically manifests during early adulthood and is seldom seen before the age of 8–10 years.^78,79^ Only three identified patients were diagnosed with ACM, all >18 years old. DCM onset usually occurs between the ages of 20 and 60 but can also affect children and the elderly.^80^ This statistic is supported by the identified DCM patients, whose reported ages at sampling ranged from 7 months to 65 years. RCM is rare, which is reflected in the number of patients included in this study (2.9%),^1^ who were 3 and 4 years old at sampling. Syndromic forms of cardiomyopathies typically manifest at a young age, which was also represented in our results.^81–83^ Strikingly, for 14.5% of patient age at sampling was not clearly defined.

Granularity of the included clinical information significantly varied among the articles. For about a third of the patients, it was only very briefly noted or non-specific, e.g. only describing a patient’s phenotype as mild or severe without supplying measurements or numerical assessments (see Supplementary material online, *Table S2*).^84^ Clinical parameters such as electrocardiogram (ECG) abnormalities, arrhythmia, echocardiographic/cardiac magnetic imaging measurements, including LV ejection fraction (LVEF), LV dilatation, and diastolic function parameters, or treatment, including the presence of an implantable cardiac defibrillator (ICD), pacemaker (PM), VAD, or HTx, were often left out, as is also illustrated in the DCM and HCM groups (*Figure 4C* and *D*). Strikingly, in 11.6% of the cases, no clinical information about the patient who provided cells for hiPSC generation was disclosed. This lack of detailed clinical information underscores the need for standardized and comprehensive reporting in future studies to improve the interpretability and clinical relevance of hiPSC-based cardiomyopathy models.

A total of 56 unique variants in 31 genes were corrected in patient-derived hiPSC lines, with 70 corrections performed in total. Corrected variants varied from being corrected in one patient to up to four patients; p.Arg403Gln in the *MYH7* gene and p.Arg14del in the *PLN* gene were rescued in hiPSCs of four patients each. For hiPSC-CM modelling in one patient, a variant in both *MYBPC3* and *MYH7* was corrected. The specifics of the corrected variants per gene are included in Supplementary material online, *Table S3*. The 56 corrected variants included intronic, missense, nonsense, frameshift, and indel variants, each with its American College of Medical Genetics and Genomics/Association for Molecular Pathology (ACMG/AMP) pathogenicity classification if listed in ClinVar.^85^ Eleven variants did not have a registered classification. Notably, among the variants registered in ClinVar, some corrected variants were classified as variants of uncertain significance (*n* = 4) or conflicting classification of pathogenicity (*n* = 6). This may be attributed to the fact that ACMG/AMP classification and ClinVar submissions are regularly updated.^86^

The final list, consisting of patient-derived variant corrections and variant introductions in donor lines, includes 91 variants across 38 genes. This compilation represents variants for which the technical feasibility of *in vitro* gene editing of human cardiomyopathy-associated variants with functional follow-up in hiPSC-CMs has been successfully demonstrated (*Table 1* as a summary of Supplementary material online, *Tables S1* and *S2*).

**Table 1:** Summary of all variants edited in human induced pluripotent stem cells either by correction or introduction

Gene	c. change	p. change
ACTC1	c.301G>A	p.Glu101Lys
ACTN2	c.740C>T	p.Thr247Met
ACTN2	c.2578C>T	p.Gln860Ter
CACNA1C	c.3343G>A	p.Glu1115Lys
DES	c.1315G>A	p.Glu439Lys
DMD	c.287del	p.Ser96Ilefs*5
DMD	c.4174C>T	p.Gln1392Ter
DMD	c.4996C>T	p.Arg1666Ter
DMD	del exons 3–7	—
DMD	del exons 8–9	—
DMD	del exon 44	—
DMD	del exons 48–50	—
DMD	del exons 48–54	—
DMD	del exon 50	—
DSG2	c.355C>T	p.Arg119Ter
DSP	c.5851C>T	p.Arg1951Ter
DSP	c.5852del13	p.R1951fs*16
DSP	c.5856del2	p.E1952fs*3
FLNC	c.7416_7418delGAA	p.Glu2472_Asn2473delinsAsp
FXN	Hyperexpansion of GAA repeats (first intron)	
GLA	c.644A>G	p.Asn215Ser
GLA	c.658C>T	p.Arg220Ter
GLA	IVS4 + c.919G>A	—
JPH2	c.482C>A	p.Thr161Lys
LAMP2	c.247C>T	p.Arg83Ter
LMNA	c.29C>T	p.Thr10Ile
LMNA	c.348dup	p.Lys117Glufs*10
LMNA	c.656A>C	p.Lys219Thr
LMNA	c.672C>T	p.Arg225Ter
LMNA	c.1621C>T	p.Arg541Cys
LZTR1	c.1739T>C	p.Leu580Pro
MIB1	c.1588C>T	p.Arg530Ter
MIB1	c.2827G>T	p.Val943Phe
MRAS	c.68G>T	p.Gly23Val
MYBPC3	c.442G>A	p.Gly148Arg
MYBPC3	c.772G>A	p.Glu258Lys
MYBPC3	c.1504C>T	p.Arg502Trp
MYBPC3	c.1800delA	p.Lys600Asnfs*2
MYBPC3	c.2372_2373insG	p.Trp792Valfs*41
MYBPC3	c.2827C>T	p.Arg943Ter
MYBPC3	c.2864_2865del	p.Pro955fs
MYBPC3	c.3217dupC	p.Arg1073Profs*4
MYH7	c.767G>A	p.Gly256Glu
MYH7	c.1063G>A	p.Ala355Thr
MYH7	c.1208G>A	p.Arg403Gln
MYH7	c.1357C>T	p.Arg453Cys
MYH7	c.1816G>A	p.Val606Met
MYH7	c.1977G>A	p.Met659Ile
MYH7	c.2129C>G	p.Pro710Arg
MYH7	c.2155C>T	p.Arg719Trp
MYH7	c.2167C>T	p.Arg723Cys
MYH7	c.2543A>G	p.Glu848Gly
MYH7	c.2555T>C	p.Met852Thr
MYH7	c.5779A>T	p.Ile1927Phe
MYLK3	c.1951-1G>T	p.P639Vfs*15
PKP2	c.1228dupG	p.Asp410fs*425
PLN	c.25C>T	p.Arg9Cys
PLN	c.40_42delAGA	p.Arg14del
PRDM16	c.559C>T	p.Glu187Ter
PRKAG2	c.905G>A	p.Arg302Gln
PRNP	c.598G>A	p.Glu200Lys
RAF1	c.770C>T	p.Ser257Leu
RBM20	c.1898C>T	p.Pro633Leu
RBM20	c.1901G>A	p.Arg634Gln
RBM20	—	p.Ser635Valfs*
RBM20	c.1906C>A	p.Arg636Ser
RTTN	c.3962G>A	p.Gly1321Asp
SCN5A	c.656G>A	p.Arg219His
SCN5A	c.665G>A	p.Arg222Gln
SCN5A	c.5693G>A	p.Arg1898His
SLC22A5	c.95A>G	p.Asn32Ser
SPEG	c.5038G>A	p.Glu1680Lys
TAFAZZIN	c.517delG	p.Asp173Thrfs*
TBX20	c.951C>A	p.Tyr317Ter
TMEM43	c.1073C>T	p.Ser358Leu
TNNI3	c.61C>T	p.Arg21Cys
TNNI3	c.508C>T	p.Arg170Trp
TNNT2	c.266T>A	p.Ile89Asn
TNNT2	c.274C>T	p.Arg92Trp
TNNT2	c.305G>A	p.Arg102Gln
TNNT2	c.421C>T	p.Arg141Trp
TNNT2	c.508GAG[3]	p.Glu173del
TNNT2	c.547C>T	p.Arg183Trp
TNNT2	c.553A>G	p.Lys185Glu
TNNT2	c.650_652delAGA	p.Lys220del
TNNT2	c.870G>T	p.Lys290Asn
TPM1	c.553C>T	p.Leu185Phe
TTL	c.655G>A	p.Gly219Ser
TTN	c.70692_70693insAT	p.Thr23565Serfs*5
TTN	c.72663del	p.Pro24223Leufs*
TTN	c.103103T>G	p.Leu34368Ter

## Discussion

While cardiovascular research is advancing rapidly through the adoption of cutting-edge editing methodologies, the resulting acceleration has created a critical gap in pivotal background data, especially regarding the application of isogenic controls. This study emphasizes the potential to strengthen *in vitro* gene editing research using isogenic control pairs through improved integration of donor and patient demographic and clinical information and calls for the development of standardized reporting practices to support this aim. Factors such as age, sex, and clinical status at the time of sampling are crucial to identify any potential biases. For instance, age can influence residual epigenetic marks in hiPSCs, such as DNA methylation, and is linearly correlated with the rate of exonic variants.^67^ Notably, companies supplying hiPSCs often do not provide information on the donor’s age or ethnicity.^18^ In addition, information on disease symptoms and their progression, family history, age at disease onset, and treatment options, including medication, and whether the patient has undergone VAD or HTx are critical factors that determine the severity of disease and aid in the classification of the patient.^1^ The availability of such clinical information is especially important in cardiomyopathies where patients with identical variants show highly variable disease phenotypes.^87^ A solid foundation of literature combining *in vitro* gene editing outcomes with patient information can help identify which patients may benefit from gene therapies and at what stage of disease progression. By comparing the clinical characteristics of patients included in successful *in vitro* gene editing experiments with those of patients in clinical trials, differences or similarities in outcomes can be better understood.

We propose that future research on hiPSC-CMs in cardiomyopathies adopts a multidisciplinary approach, integrating a comprehensive set of clinical parameters tailored to the specific type of cardiomyopathy. This proposal is based on the known limitations of hiPSC-CMs when studied in isolation and the recognition that more robust disease modelling requires the integration of complementary expertise. Combining genomics, molecular and cell biology, bioengineering, computational approaches, and clinical cardiology is expected to improve physiological relevance and support the translation of findings towards clinical application. Key parameters should include donor characteristics such as age at sampling and sex, alongside clinical parameters such as disease severity, ECG findings, presence of arrhythmia and/or conduction disturbances, left and/or right ventricular dilatation and ejection fraction, echocardiography and cardiac magnetic resonance (CMR) imaging findings, age at disease onset, medication status at sampling, and treatment history. Additionally, cardiomyopathy-specific parameters, such as the Task Force Criteria, established diagnostic guidelines for ACM or wall thickness measurements for HCM, should also be taken into account.^1,88^ Based on the findings from our systematic review, we propose a (minimal) set of recommended parameters in *Table 2* for standardized use across the cardiomyopathy field and beyond. At present, there is no clear consensus on which clinical parameters should be prioritized for integration, particularly given the progressive and heterogeneous nature of cardiomyopathies. Clinical data can range from raw diagnostic imaging and electrophysiological measurements to interpretative summaries and diagnostic codes, each offering varying levels of granularity. Since cardiomyopathies often evolve over time, relying solely on single-time point assessments may limit the fidelity of disease modelling. Instead, incorporating longitudinal markers of disease severity, such as the lowest recorded LVEF or other quantitative indicators of cardiac dysfunction, may provide more accurate correlates for *in vitro* modelling of severe phenotypes. Moreover, functional outputs from hiPSC-CM models, including ECG-like contraction patterns observed in e.g. engineered cardiac organoids,^89^ can be aligned with patient-level endpoint data to further enhance clinical comparability and predictive value.

**Table 2:** Proposed reporting framework for studies on *in vitro* gene editing in cardiomyopathy research

hiPSC line	Name, identifier
**Donor information**	Sex
	Age at sampling
	Sampling source
	Medication use prior to sampling (e.g. anaesthetics)
**Genetic**	Gene and gene variant
	HGVS nomenclature
	ACMG/AMP classification for variant pathogenicity
	Variant zygosity
**Clinics**	Cardiomyopathy diagnosis (and subtype) with criteria fulfilment
	Symptom onset
	Disease severity [classification, disease stage, and specific parameters (e.g. left ventricular hypertrophy (LVH) for HCM and left ventricular end-diastolic diameter (LVEDD) and LVEF for DCM)]
	ECG and cardiac imaging findings (+ age)
	Gene-specific manifestations (e.g. atrioventricular block (AV) block for *LMNA* DCM and low-voltage ECG for *PLN* DCM)
	Major cardiac events [including e.g. hospitalization and HF (+ age)]
	Interventions [including e.g. ICD (+ age)]
	Non-cardiac symptoms
	Comorbidities
	Medication history
	Lifestyle factors
**Reprogramming and gene editing**	Method
	Genetic integrity assessment
	Pluripotency potential
	Additional characterization
	Gene editing quality control
**hiPSC-CM information**	Media composition
	Duration of culturing
	Surface and substrate
	Characterization
	Quality control
	Purity enrichment
	Maturation

hiPSC, human induced pluripotent stem cell; hiPSC-CM, human induced pluripotent stem cell-derived cardiomyocyte; HGVS, Human Genome Variation Society; ACMG/AMP, American College of Medical Genetics and Genomics/Association for Molecular Pathology.

Inclusion of such parameters raises important ethical considerations, such as determining which diseases and genes should be prioritized and the criteria that should be met for making these decisions. Establishing priority diseases requires careful evaluation of numerous factors, and there is no simple answer to those challenges. Furthermore, gene editing methods are primarily used for monogenic diseases, and many cardiac diseases are oligogenic or do not have a known genetic cause, although new approaches based on multiplexed CRISPR systems aiming to simultaneously target and correct multiple genetic variants are being developed.^90^ In this review, we reported on symptomatic cardiomyopathy patients. Nevertheless, *in vivo* gene editing holds promise to treat both symptomatic patients and pre-symptomatic patients with high-risk variants, offering a preventive approach. Nevertheless, this remains a challenging approach due to the large phenotypic heterogeneity among (a)symptomatic patients. It is important to provide a detailed phenotypic description of the patients, as the phenotype may become irreversible in advanced stages of cardiac disease.^1^ Moreover, such descriptions may provide crucial insights to explore reasons for variable disease courses in patients with identical pathogenic variants. Furthermore, we have categorized the existing literature by cardiomyopathy subtype, noting that the relative focus on HCM and DCM in isogenic hiPSC-CM studies appears to broadly reflect their higher prevalence in the general population, whereas rarer subtypes such as NDLVC (recognized only in 2023^1^) and syndromic cardiomyopathies remain significantly underrepresented.

The presented work has some limitations related to our search criteria and the broader challenges of synthesizing findings from published studies. Relevant studies or data may have been excluded due to inconsistencies in how information is reported across publications. The current structure of scientific articles often hinders systematic screening, with essential details frequently moved to supplementary materials or omitted entirely. Our inclusion criteria were limited to symptomatic cardiomyopathy patients, excluding individuals harbouring pathogenic variants who remained asymptomatic. While this approach ensured a focus on clinically relevant phenotypes, it may have omitted valuable insights into early or subclinical disease stages. Another limitation concerns the characterization of gene-edited iPSC clones in the included studies. Although our review focused on the use of isogenic lines to control for genetic background, most of the assessed articles relied on a single edited clone and provided only basic clone-level characterization. Best practices in iPSC gene editing generally encourage generating multiple independent clones, verifying pluripotency with several markers, evaluating off-target events, and excluding clones carrying unintended genomic alterations. Given the well-documented variability between iPSC clones and the potential for off-target effects introduced by gene editing tools, insufficient reporting of these validation steps constrains the interpretability, robustness, and generalizability of variant-specific phenotypes. A further limitation relates to the therapeutic modalities included in this review. Our analysis focused specifically on mutation-targeted genome editing approaches applied in isogenic hiPSC models and therefore excluded studies using gene knockdown or exogenous gene delivery strategies. Although these approaches, such as antisense oligonucleotide-mediated exon skipping, RNA interference, and viral gene replacement, are further along the translational pipeline for several cardiomyopathies, they operate through mechanisms distinct from variant-specific correction and fall outside the defined scope of this review. Nonetheless, their omission restricts the breadth of the therapeutic landscape presented here. To provide context for clinical translation, we note that these complementary strategies remain highly relevant and should be considered alongside mutation-specific genome editing in future integrative assessments of cardiomyopathy gene editing therapies. Additionally, we only included studies that performed functional testing in hiPSC-CMs, which reflects our emphasis on experimentally validated data. However, this does not imply that the genetic variants assessed in the excluded studies should not be considered for future clinical trials on gene editing therapies. It is also worth noting that we did not systematically report specific outcomes from functional studies, as the field is still progressing towards refining the physiological characteristics of hiPSC-CMs. These models continue to evolve in their maturity and reliability, which impacts the interpretability and generalizability of the results. To address these challenges, we advocate for standardized reporting practices that ensure critical experimental and patient-specific details are consistently documented alongside experimental data. This will enhance the usability of findings in both research and clinical trial design.

Working with stem cells raises multiple ethical considerations. The use of hiPSCs has largely replaced that of human embryonic stem cells (ESCs), addressing many ethical concerns.^91^ Therefore, obtaining informed consent from patients donating cells for hiPSC generation remains paramount. Patients and donors should be informed about the specific cellular source, methods, and potential risks associated with cell collection and whether the cells will be used solely for diagnostic and therapeutic purposes or for research and commercialization as well.^18^ Additionally, should *in vivo* gene editing therapy enter clinical practice, patients must be informed about the latest knowledge regarding long-term efficacy and risks associated with hiPSC-based treatments. This presents a communication challenge, requiring both researchers and clinicians to convey complex information in a way that aligns with each patient’s level of understanding.^92^ Both patient and healthcare professional perspectives and acceptance are crucial when implementing novel diagnostic tools and treatments.^93,94^ Acceptance of cell- and gene-based therapies, including gene (genome) editing, increases after information is provided.^94,95^ Additionally, disease severity seems to be positively correlated with the acceptance of stem cell research and the perception that it is morally acceptable and advantageous for healthcare and society.^96^ Ongoing ethical challenges include managing donor consent over time, restrictions on cell use, recontacting donors for updated consent, and safeguarding privacy given the intrinsic traceability of hiPSC lines.^97^ Furthermore, regulatory challenges include managing immune responses, ensuring long-term safety and efficacy monitoring, navigating complex approval procedures, establishing good manufacturing practice (GMP)-compliant manufacturing, and addressing the high financial, logistical, and multidisciplinary demands required for successful clinical translation of genome editing therapies.^98^

## Conclusions and future directions

In conclusion, we provide a comprehensive overview of studies using isogenic control pairs for *in vitro* gene editing in hiPSC-CMs for cardiomyopathy research. Our systematic analysis highlights a recurrent gap: many studies fail to provide essential details on cardiomyopathy diagnosis and the severity of the condition in the individuals from whom the cells were derived. This lack of information likely extends beyond isogenic cells created through gene editing for cardiomyopathy research. The current state of reporting hinders comparative meta-analyses of the individual studies and limits the reproducibility and generalizability of findings. Structured and detailed reporting is essential to establish a robust foundation for future clinical trials utilizing gene editing for cardiomyopathies. The sooner the field adopts standardized reporting practices, the faster translation to clinic applications can be facilitated. To enable this clinical translation of genome editing for cardiomyopathies, several key technological and methodological advances are required. Technologically, more efficient and CM-specific delivery systems, such as optimized adeno-associated viruses (AAVs) or non-viral nanoparticles, are essential to ensure targeted and safe *in vivo* editing. Additionally, next-generation tools such as base editors and prime editors are being developed to improve editing precision and minimize off-target effects. Scalable editing strategies that function effectively in adult human cardiac tissue are also critical, as are robust *in vivo* validation platforms using large animal models that better recapitulate human cardiac physiology. Methodologically, this field requires standardized pre-clinical testing pipelines to assess editing efficiency, specificity, and long-term cardiac safety. Approaches that support multiplex gene editing are particularly important, given the oligogenic nature of many cardiomyopathies. Improved variant interpretation frameworks and gene curation efforts, such as those led by ClinGen, will help prioritize clinically actionable targets. Furthermore, integrating genetic and phenotypic data will be essential for stratifying patients who are most likely to benefit from gene editing interventions. Furthermore, we strongly encourage the active involvement of patients and clinicians in the pre-clinical part of clinical trials. This engagement will allow for discussions on the ethical and practical aspects of gene editing approaches ensuring that they align with patient needs and expectations.

## Lead author biography


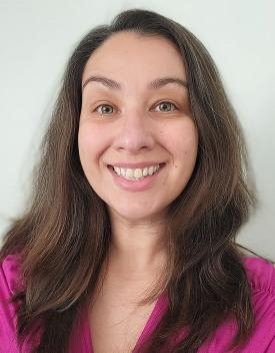


Dr Magdalena Harakalova is a translational cardiovascular epigeneticist at the University Medical Center Utrecht. She holds a visiting scientist appointment at Maastricht University. Her research centres on understanding the molecular basis of prevention of inherited cardiomyopathies, with a focus on understanding disease modifiers. She combines high-throughput sequencing, functional assays, and stem cell and gene editing models to evaluate pathogenic mechanisms and improve variant interpretation. She contributes to multidisciplinary efforts aimed at translating genomic findings into improved diagnostics and clinical care for patients with rare cardiac conditions.

## Supplementary Material

oeaf161_Supplementary_Data

## Data Availability

All datasets underlying the results of this study are contained in the article and its supplementary files. The PubMed search output file used for the systematic search and the scoring sheet for inclusion criteria are available from the corresponding author upon request.
