# Supplementary material for: Setting the stage for cardiomyopathy gene editing trials: a systematic review of isogenic pair use in human induced pluripotent stem cell-derived cardiomyocyte research
Source: Eur Heart J Open. 2025 Dec 3;6(1):oeaf161. doi: 10.1093/ehjopen/oeaf161 (PMC12796640; doi:10.1093/ehjopen/oeaf161)
Supplement: oeaf161_Supplementary_Data [file oeaf161_supplementary_data.zip › Figure_S1.pdf]

PRISMA 2020 flow diagram for new systematic reviews which included searches of databases and registers only

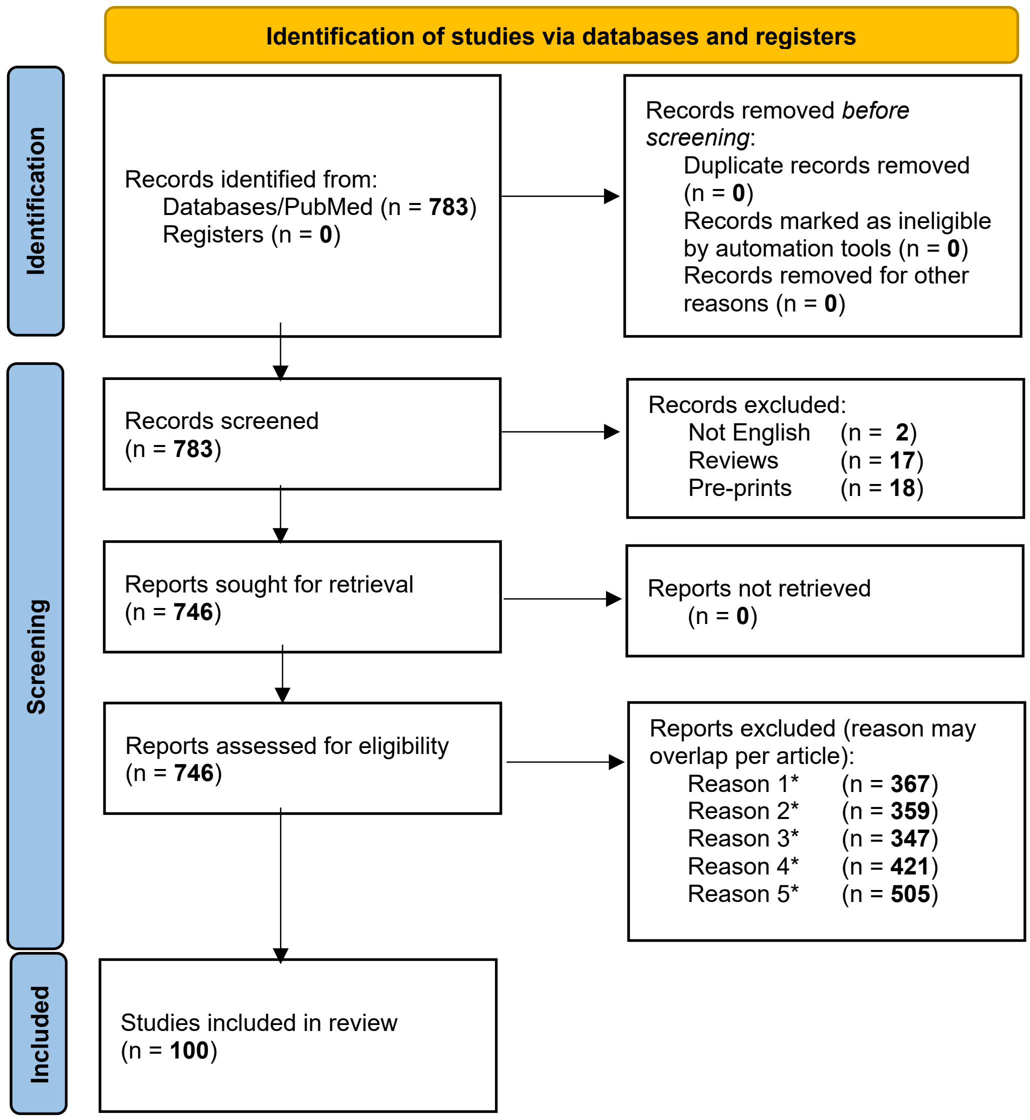

***Reason 1:** Not fulfilling Inclusion criterion #1: “Reported human (pathogenic) variants associated with patient cardiomyopathy phenotype either in the cell-donating patient (variant correction) or in previously reported patients (variant introduction)”

***Reason 2:** Not fulfilling Inclusion criterion #2: “Cardiomyopathy diagnosed clinically and caused by a pathogenic variant either in the cell-donating patient (variant correction) or in previously reported patients (variant introduction).”

***Reason 3:** Not fulfilling Inclusion criterion #3: “Correction and/or introduction of a pathogenic variant in a hiPSC cell line using gene editing.”

***Reason 4:** Not fulfilling Inclusion criterion #4: “Differentiation into cardiomyocytes (hiPSC-CMs)”

***Reason 5:** Not fulfilling Inclusion criterion #5: “Functional measurements in the differentiated hiPSC-CMs isogenic pair, including live cell measurements, such as electrophysiology, contractility, or calcium handling.”

Source: Page MJ, et al. BMJ 2021;372:n71. doi: 10.1136/bmj.n71.

This work is licensed under CC BY 4.0. To view a copy of this license, visit https://creativecommons.org/licenses/by/4.0/
